# Cutaneous reaction to cancer treatment: Atezolizumab-induced lichen planus pemphigoides

**DOI:** 10.1016/j.jdcr.2025.06.015

**Published:** 2025-06-23

**Authors:** Hira Asim, Cody Tilton, Julie Hancock, Jesse Fike

**Affiliations:** a Long School of Medicine, The University of Texas Health Science Center at San Antonio, San Antonio, Texas; b Department of Dermatology, The University of Texas Health Science Center at San Antonio, San Antonio, Texas

**Keywords:** atezolizumab, immunotherapy, inhibitors, lichen planus pemphigoides, programmed cell death ligand 1

## Introduction

Lichen planus pemphigoides (LPP) is a rare autoimmune dermatosis with overlapping features of lichen planus (LP) and bullous pemphigoid (BP). Although it was previously believed to be a variant of these conditions, it has been recently suggested that LPP is a separate entity. The clinical features of LPP include lichenoid papules and plaques admixed with subepidermal blisters commonly affecting the upper and lower extremities with additional involvement of the nails and oral mucosa. Autoantibodies against type XVII collagen (BP180) lead to the deposition of IgG antibodies and complement factor C3 at the dermoepidermal junction. It has been hypothesized that initial lichenoid lesions and inflammation precipitate the development of these autoantibodies.^1^ Although LPP has been associated with viral infections (varicella and hepatitis B), malignancies, and drugs (especially angiotensin-converting enzyme inhibitors), there have been a few reported cases of programmed cell death ligand-1 (PD-L1) inhibitor-induced LPP.^1^^,^^2^ Atezolizumab is an anti-PD-L1 agent that is indicated for use in treating several types of malignancies such as non-small cell lung cancer and urothelial cancer.^3^ We present a case of atezolizumab-induced LPP in a patient being treated for hepatocellular carcinoma.

## Case report

**Fig 1:**
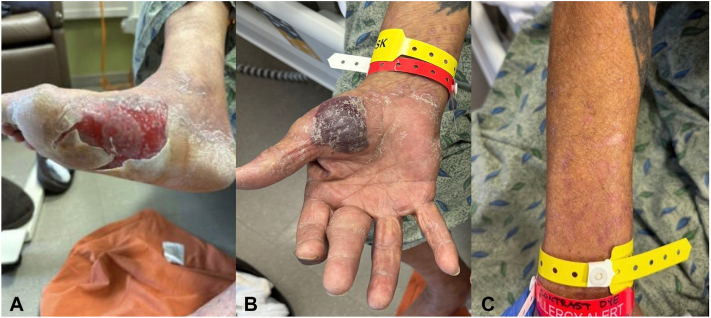
Physical examination findings at presentation including tense and ruptured bullae on the palms and soles as well as purple, flat-topped papules on the forearms.

A 70-year-old man with a history of hepatocellular carcinoma (HCC), cirrhosis, and treatment-naive hepatitis C diagnosed 6 years prior presented to the hospital for a progressive painful, blistering rash of 3 weeks duration. He began receiving atezolizumab immunotherapy for HCC 6 months prior to the onset of the rash, with the most recent cycle completed 1 month prior to onset. Of note, he also had started receiving radiation therapy to the liver around the same time the rash began in areas that were not treated with radiation. At another facility, he had been prescribed multiple courses of antibiotics including trimethoprim-sulfamethoxazole and cephalexin, intramuscular methylprednisolone, a course of oral prednisone, and topical steroids to treat the rash, none of which provided relief. Physical examination revealed a tense, hemorrhagic bulla to the right palm, ruptured blisters with painful erosions to the bilateral soles, and purple, flat-topped papules to the forearms (Fig 1). He had hemorrhagic vesicles and erosions to the hard palate, buccal mucosa, and lateral tongue but no ocular or genital involvement.

**Fig 2:**
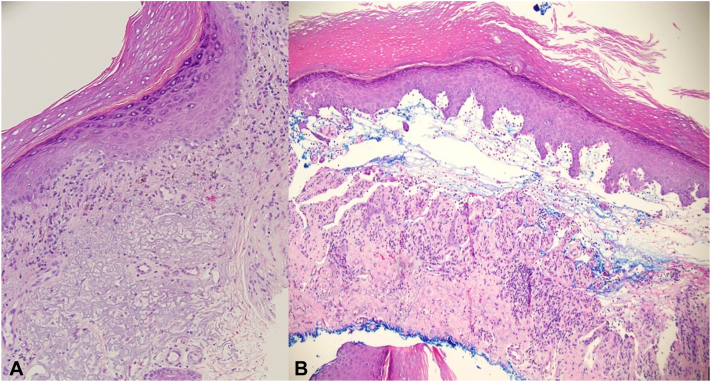
**A,** Biopsy with H&E revealing compact orthokeratosis, wedge-shaped hypergranulosis, saw-tooth rete ridges, and lichenoid dermatitis. **B,** Biopsy with H&E revealing subepidermal split with lymphocytes and eosinophils (**A** and **B,** Hematoxylin-eosin stain; original magnifications: **A,** ×20; **B,** ×10.)

Initial differential diagnosis included bullous LP, LPP, BP, paraneoplastic pemphigus, bullous-fixed drug eruption, IgA bullous dermatosis, epidermolysis bullosa acquisita, porphyria cutanea tarda, and bullous lupus. Three shave biopsies were obtained: a lichenoid papule on the forearm for hematoxylin and eosin (H&E), a partial biopsy of the palmar bulla for H&E, and perilesional skin adjacent to the palmar bulla for direct immunofluorescence (DIF). On H&E, both the forearm papule and palmar bulla revealed LP-like changes including compact orthokeratosis, wedge-shaped hypergranulosis, saw-tooth rete ridges, and lichenoid dermatitis (Fig 2, *A*). The palmar bulla H&E additionally revealed a subepidermal split with lymphocytes and eosinophils (Fig 2, *B*). DIF showed linear deposition of C3 and IgG along the basement membrane.

The patient’s history, physical examination, and histopathologic findings were consistent with LPP, most likely induced by his atezolizumab immunotherapy. The patient was prescribed prednisone 1 mg/kg daily, doxycycline, and niacinamide with initial improvement of his lesions and symptoms. He was also prescribed topical clobetasol to use twice daily. After a discussion with his oncologist, atezolizumab was held with plans to switch to an alternative immunotherapy. Unfortunately, the patient passed away from HCC-related complications shortly after our evaluation.

## Discussion

LPP is a rare autoimmune blistering disorder with clinical and histopathologic features of LP and BP. The development of anti-BP180 autoantibodies, potentially as a consequence of lichenoid inflammation, has been proposed as a major mechanism in the pathogenesis of LPP.^4^ With the advent of PD-L1 inhibitors for the treatment of solid organ and hematologic malignancies, there have also been discoveries of the drug’s nonspecific activation of the immune system, contributing to cutaneous adverse effects such as vitiligo, pruritus, lichenoid dermatitis, psoriasiform eruptions, and immunobullous dermatoses.^5^

Our patient’s clinical presentation consisted of tense and ruptured bullae, purple, flat-topped papules, and hemorrhagic vesicles and erosions. The histopathological evidence included compact orthokeratosis, wedge-shaped hypergranulosis, saw-tooth rete ridges, lichenoid dermatitis, and subepidermal split with lymphocytes and eosinophils. DIF revealed the deposition of linear C3 and IgG along the basement membrane. The combination of these findings is most consistent with a diagnosis of LPP. Previous literature has described the eruption of BP (tense bullae and vesicles possibly with mucosal involvement) as well as lichenoid reactions (flat-topped polygonal papules/plaques and visible Wickham striae and/or hypertrophic vesiculobullous lesions) in patients treated with anti-PD1 and PD-L1 inhibitor therapy.^6^, ^7^ LPP has also been reported with these therapies, but more rarely. Although it is possible that our patient had a combination of 2 distinct disorders (BP and LP), we believe that LPP is the most accurate and comprehensive diagnosis.

The time course of these eruptions can be variable, but the timeline in our patient does fit within the timelines described in the literature. A systematic review of cutaneous adverse reactions to anti-programmed cell death protein 1 inhibitor treatment (specifically nivolumab and pembrolizumab) postulated that the average time of onset for bullous eruptions after the initiation of immunotherapy is approximately 3 months but can range from 3 weeks to 21 months. It has also been proposed that the latency interval between the first dose of anti- programmed cell death protein 1 inhibitor treatment and the onset of a lichenoid reaction can range from a few days to several months.^6^ Our patient began experiencing a bullous, lichenoid rash approximately 7 months after atezolizumab was initiated and approximately 2 months after the last administration, a latency period consistent with the timeframe described in the literature review.

We must consider the limitations that prohibit the establishment of a clear causal relationship between this patient’s adverse reaction and the atezolizumab therapy, including the potential for hepatitis C-related cutaneous effects, paraneoplastic effects triggered by the patient’s history of HCC, involvement of radiation in the cancer treatment plan, and the lack of further diagnostic exploration due to the patient’s death. This case report exemplifies the need for further study into the specific association between PD-L1 inhibitors and LPP, as it has not been well-documented. Thus, future collaborations between dermatologists, dermatopathologists, and oncologists should be encouraged to prevent and manage the onset of serious cutaneous side effects while effectively treating malignancies.

## Conflicts of interest

None disclosed.
